# The Potential of Intrinsically Magnetic Mesenchymal Stem Cells for Tissue Engineering

**DOI:** 10.3390/ijms19103159

**Published:** 2018-10-14

**Authors:** Fransiscus F. A. Kerans, Lisa Lungaro, Asim Azfer, Donald M. Salter

**Affiliations:** Centre for Genomics and Experimental Medicine, MRC IGMM, University of Edinburgh, Edinburgh EH4 2XU, UK; s1578984@sms.ed.ac.uk (F.F.A.K.); Lisa.Lungaro@gmail.com (L.L.); aazfer@ed.ac.uk (A.A.)

**Keywords:** mesenchymal stem cells, magnetic nanoparticles, magnetotactic bacteria, magnetosomes, tissue engineering

## Abstract

The magnetization of mesenchymal stem cells (MSC) has the potential to aid tissue engineering approaches by allowing tracking, targeting, and local retention of cells at the site of tissue damage. Commonly used methods for magnetizing cells include optimizing uptake and retention of superparamagnetic iron oxide nanoparticles (SPIONs). These appear to have minimal detrimental effects on the use of MSC function as assessed by in vitro assays. The cellular content of magnetic nanoparticles (MNPs) will, however, decrease with cell proliferation and the longer-term effects on MSC function are not entirely clear. An alternative approach to magnetizing MSCs involves genetic modification by transfection with one or more genes derived from *Magnetospirillum magneticum* AMB-1, a magnetotactic bacterium that synthesizes single-magnetic domain crystals which are incorporated into magnetosomes. MSCs with either or *mms6* and *mmsF* genes are followed by bio-assimilated synthesis of intracytoplasmic magnetic nanoparticles which can be imaged by magnetic resonance (MR) and which have no deleterious effects on MSC proliferation, migration, or differentiation. The stable transfection of magnetosome-associated genes in MSCs promotes assimilation of magnetic nanoparticle synthesis into mammalian cells with the potential to allow MR-based cell tracking and, through external or internal magnetic targeting approaches, enhanced site-specific retention of cells for tissue engineering.

## 1. Introduction

Due to their unique physical properties, magnetic nanoparticles (MNPs) are being increasingly recognized as having potential in a range of biomedical applications either alone or through cell-based therapies [1]. They are typically composed of a magnetite (Fe_3_O_4_) or maghemite (γ-Fe_2_O_3_) core. Both are naturally ferromagnetic in bulk, being permanently attracted to magnets or are permanently magnetic, but at diameters smaller than their intrinsic superparamagnetic radius and greater than their single domain radius, they become superparamagnets. In general, iron oxide MNPs smaller than 30 nm are superparamagnetic, i.e., their magnetization only occurs in the presence of an external magnetic field and are termed superparamagnetic iron oxide nanoparticles (SPIONs). The magnetic responsiveness of MNPs allows them to be identified by magnetic resonance (MR) imaging and as such they are useful for tracking the destination and fate of loaded cells such as stem cells used in tissue engineering applications [2]. The use of internal or external magnetic forces following in vivo administration of magnetic stem cells may also allow targeting to specific sites of interest such as areas of tissue damage improving efficacy of treatments [3]. In this review, we look at the potential for the use of genetically modified magnetic mesenchymal stem cells for tissue engineering.

## 2. Extrinsic Magnetization

Finding the optimal method to magnetize human mesenchymal stem cells (MSCs) for tissue engineering or other cell-based therapies has proved challenging. Unmodified SPIONs are readily taken up by phagocytic cells such as macrophages but this contrasts to the relatively poorer ability of non-phagocytic cells such as MSCs to ingest extrinsically applied MNPs and the possibility of adverse effects on MSC proliferation and migration [1]. A number of strategies have been developed to increase uptake of MNPs by non-phagocytic cells such as MSCs. These include coating of the MNPs with a range of substances including surfactant, precious metals, silica, carbon coatings, cellulose, and chitosan. Currently, three prominent SPION coating types—polyethylene glycol (PEG), starch, and dextran—appear to be most beneficial and generally used [2,3] with ferumoxide, ferucarbotran, and Feridex SPIONs coated with being approved for human use. Cellular uptake of MNPs is by endocytosis but may be enhanced by the use of agents traditionally used for gene transfection such as Fugene, Superfec and Lipofectamine [4]. Furthermore, cell labelling with transfection agents has saturation effects, requiring a high concentration of transfection agents and a long incubation period, which may have a negative effect on cell function after transplantation [5]. However, uptake of MNPs by MSCs is variable over a population and cell content is likely to decrease following proliferation of administered MSCs in vivo. Nanoparticles may also be released from cells and thereafter drain to lymphatics or be taken up by neighboring tissue cells. Potential detrimental effects on cell proliferation and migration [1] are continually debated with more recent reports indicating less adverse effects on cell behavior [6,7,8,9], although their long-term in vivo effects need to be fully evaluated. Nevertheless, there is a significant body of literature indicating that metallic nanoparticles have cytotoxic potential with SPION accumulation affecting cell differentiation and inducing oxidative stress to cells [10]. The impact of SPIONs on MSC function, such as proliferation, immunomodulation, and differentiation, are being explored. Ferucarbotran, a SPION with a crystalline nonstoichiometric Fe^2+^ and Fe^3+^ iron oxide core, stimulates in vitro MSC proliferation [11,12,13], whereas other iron oxide nanoparticles have been shown to enhance osteogenic differentiation in human bone-derived mesenchymal stem cells (hBMSCs) in vitro [14]. More work is clearly required in this area to establish the effects of different MNPs over a range of concentrations on specific MSC function and differentiation.

## 3. Intrinsic Magnetization

### 3.1. Magnetotactic Bacteria and Magnetosomes

To overcome at least some of the above problems, there has been recent interest in the generation of human MSCs capable of synthesizing de novo magnetic nanoparticles based upon processes used by magnetotactic bacteria (MTB). MTB are Gram-negative organisms, initially identified by Salvatore Bellini, which are able to align with external magnetic fields. This ability is given by chains of linearly arranged subcellular magnetic organelles, called magnetosomes [15,16,17,18]. Magnetosomes are lipidic membranes, originating from MTB cytoplasmic membrane [19,20] containing crystals of either magnetite (Fe_3_O_4_), greigite (Fe_3_S_4_), or maghemite (γ-Fe_2_O_3)_. The length of the magnetosome crystal iron oxide core, determined by Transmission Electron Microscopy (TEM) investigation, measures between 5 and 120 nm. *Magnetospirillum magneticum* AMB-1 and *Magnetospirillum gryphiswaldense* MSR-1 strains are characterized by magnetosomes of cubo-octahedral geometry, sized between 30 and 50 nm [21]. This size makes magnetosomes single-domain crystals with the maximum possible magnetic moment per unit volume. In *Magnetospirillum* AMB-1 and *Magnetospirillum* MSR-1, there is only one magnetosome chain composed by 15–30 magnetosomes, while the *Magnetobacterium bavaricum* strain shows more chains, each of them consisting of more strands of magnetosomes [22].

**Figure 1:**
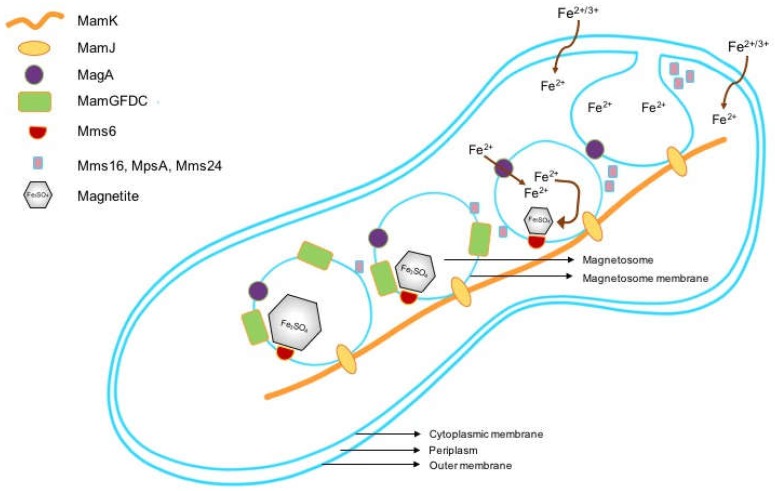
Sequential steps in the formation of magnetosomes in magnetotactic bacteria.

Magnetosome formation is a multi-step process involving invagination of vesicles from the inner membrane; sorting and targeting of magnetosome membrane proteins to the forming structure; iron transport; crystallization of magnetite crystals; assembly, as well as positioning of formed invaginations into a chain-like structure (Figure 1). 

Essential genes for the biosynthesis of magnetosomes in MTB are clustered predominantly in large genomic units called the magnetosome gene island (MAI) [15]. This conserved region contains a number of operons including magnetosome membrane (*mam*) *AB* (17 genes), *mamGFDC* (4 genes), and *mamXY* (4 genes), the magnetic particle membrane-specific (mms) operon *mms6* (5 genes) and the monocistronic *mamW* operon [23]. Without MAI or the operon *mamAB*, there is no magnetic phenotype [24,25]. Studies have shown that the large *mamAB* operon is predominantly involved in iron transport, magnetosome membrane biogenesis, and crystallization of magnetite particles, as well as their chain-like organization and intracellular positioning, whereas the smaller *mamGFDC*, *mms6* and *mamXY* operons are mainly associated with the formation properly sized and crystals morphology [19].

Magnetosome membrane formation from the inner cell membrane is important magnetosome biomineralization acting as a “nano-reactor” for crystal formation [26] by facilitating the creation of the chemical conditions required for the biomineralization and protection against cytotoxicity. Magnetosome vesicles remain as invaginations along the entire mineralization process, being initially empty or, in latter stages, containing iron. The formation of vesicles appears to be a necessary condition for the biomineralization process although vesicles may be seen as MTB in iron-deprived conditions [24,27]. Magnetosome proteins are usually targeted to the magnetosome membrane or to the cytosolic side. MamA, a highly conserved magnetosome-associated protein, acts as a multiprotein interaction site, forming homo-oligomers with central pore cavity [28]. MamE, predicted to be an integral membrane protein containing a transmembrane region, has a role in protein sorting and initiation of biomineralization [29], a stepwise process involving nucleation, growth, and regulation of crystal morphology [30]. Iron accumulation in magnetosome membrane vesicles appears to occur subsequently to iron uptake [26] and formation of magnetite is favored within a narrow redox range with a 2/1 ratio of Fe^3+^/Fe^2+^. MagA is a putative iron transporter [31] but recent genetic experiments have shown that magnetosomes in *magA*-deletion mutants resemble those of wild-type cells [32]. 

Complete understanding of crystal growth and regulation within magnetosomes is ongoing but it is clear that the process is tightly controlled. Some proteins, even if not directly involved in the magnetosomes biomineralization process, play a role in the determination of magnetosome size and morphology [33]. Genetic studies have shown that smaller magnetite crystals are formed in mutants that lack MamS, MamR, MamN, MamF, Mms-F, Mms5, Mms6, or Mms7. Mms5, Mms6, Mms7, and Mms13 have been identified as being in the magnetite biomineralization process. Mms proteins localize to the magnetosome membrane and are located on the surface of synthesized cubo-octahedral crystals synthesized in Magnetospirillum magneticum strain AMB-1 [30,34,35]. Mms7 plays key roles in the control of magnetite crystal growth and morphology of magnetite crystals in MTB [36]. Both *mms6* and *mmsF* are contained within the *mms6* operon and their contribution to magnetosome biogenesis is beginning to be understood [37]. Mms6 differs from other Mms proteins as it presents a negative charge at neutral pH, the others being positively charged [34]. The sequence of the *mms6* gene shows a high consensus among different MTB species [38], especially in the C-terminal region. The mass of the protein coded by *mms6* is 12–15 kDa, somewhat larger than the 6 kDa Mms6 protein identified by SDS/PAGE, suggesting that Mms6 undergoes post-transcriptional protease cleavage [34,39]. The predicted secondary structure of Mms6 is that of an unstructured N-terminal domain, a transmembrane helix and a C-terminal which may form an α-helix structure [15]. The presence of the helix is supported by protein model structure and sequence analysis, while 3D models show a negative patch on its carboxy-terminal domain (CTD) that is believed to be an iron binding site [15]. The Mms6 protein is embedded on the interior of the magnetosome membrane and is tightly associated with the formed mineral. Mms6 promotes the formation of uniform isomorphic superparamagnetic magnetite nano-crystals and helps regulate the crystal morphology of magnetite [25]. Possible roles for Mms6 in recruitment of other Mms proteins to the magnetosome and in magnetite crystal nucleation have also been proposed [35,40,41].

Significantly, recombinant Mms6 binds iron and, using either room temperature co-precipitation or partial oxidation of ferrous hydroxide, aids formation of magnetite particles in vitro that are similar to those of magnetosomes [34,37]. Analysis of gene deletion mutants and transformants of *Magnetospirillum magneticum* AMB-1 expressing partially truncated Mms6 protein reveals that deletions in the N-terminal or C-terminal regions disrupt proper protein localization to the magnetite surface, resulting in a change in the crystal morphology [42]. Staniland and Rawlings have recently proposed a mechanism for the function of Mms6 as a specific magnetite nucleation protein and have highlighted key features for this action including self-assembly to display a charged surface for specific iron binding, with the curvature of the surfaces determining the particle size [38].

Less is known regarding the function of MmsF. In *M. Magneticum* AMB-1, *mmsF* is located directly downstream to *mms6*, separated from it by only 17 nucleotides. No promoter is identified upstream of *mmsF*, so *mms6* and *mmsF* are possibly co-transcribed [43]. MmsF is an α-helical membrane spanning protein. It self-assembles in vitro into 36 nm diameter spherical aggregates that control nanoparticle size and magnetite homogeneity in chemical precipitation reactions, supporting its indicated in vivo role as a master regulator of magnetite biomineralization and nanoparticle morphology [44]. 

### 3.2. Formation of Magnetosome-Like Nanoparticles in Mammalian Cells

As yet, relatively few magnetosome-associated genes have been tested in mammalian cell systems, with the limited number of published studies having focused on *magA* or *mms6*. *magA* from two different MTB has been studied as a potential reporter gene for Magnetic Resonance Imaging (MRI), whereas *mms6*, and to a lesser extent *mmsF*, have been assessed for nanoparticle production in human mesenchymal stem cells.

Transgenic studies using the *magA* gene isolated from AMB-1 MTB demonstrated that stable transfection of the human embryonal kidney cell line and a mouse neuroblastoma N2A cell line led to deposition of intracellular iron that could function as a MRI reporter [45,46]. Accumulation of intracellular iron was dependent on iron supplementation and gene expression. Produced nanoparticles of around 250–450 nm were found throughout the cytoplasm of cells but were most easily identified within membrane-enclosed structures [45]. Magnetic separation and X-ray powder diffraction indicated that the particles were magnetic and consisted primarily of magnetite. *mms6* has also been shown to act as a MR reporter when transfected into a cancer cell line, with *mms6*-expressing cells forming clusters of nanoparticles within and outside membrane-enclosed structures [47].

Unfortunately, in these studies, cancer cell lines or embryonal kidney cells, rather than stem cells, were studied and magnetic behavior of the particles produced by the transfected cells was not demonstrated by superconducting quantum interference device (SQUID) magnetometry. Nevertheless, these studies have been important in demonstrating the potential of a transgenic approach to magnetizing MSCs for clinical purposes.

### 3.3. Biosynthesis of Magnetic Nanoparticles by Human Mesenchymal Stem Cells

**Figure 2:**
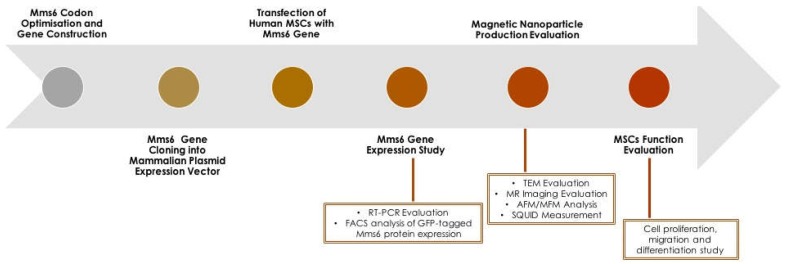
Workflow for induction and assessment of magnetic nanoparticle production in human mesenchymal stem cells (MSCs) following *mms6* gene transfection.

Recent studies by our group have shown the potential of using a transgenic approach to magnetize MSCs by transfection with either or both the *mms6* and *mmsF* genes [48] derived from *Magnetospirillum magneticum* AMB-1, a magnetotactic bacterium that synthesizes single-magnetic domain crystals which are incorporated into magnetosomes. These studies involved transfecting human MSCs with modified *mms6* or *mmsF* genes, after which gene expression studies, assessment of nanoparticle production and effects on MSC function were established (Figure 2). 

The *Magnetospirillum magneticum* AMB-1 *mms6* and *mmsF* DNA bacterial sequences were codon-optimized for mammalian expression and a Kozak sequence added, then synthesized. The optimized sequence was synthesized and cloned in their proprietary vector with SacI and KpnI restriction sites. The synthetic genes were then cloned into a pcDNA3.1 expression vector at BamH1 and EcoRI restriction sites. The pcDNA3.1 expression vectors were transfected either alone or together as separate plasmids into human adipocyte-derived MSCs with either X-tremeGENE HP or FugeneHD. The transfected MSCs were cultured with the addition of 3.4 µL/mL of ferric quinate solution to a final concentration of 34 µM, ferric quinate being used as standard as a source of iron for magnetobacterial culture and magnetosome formation [49].

**Figure 3:**
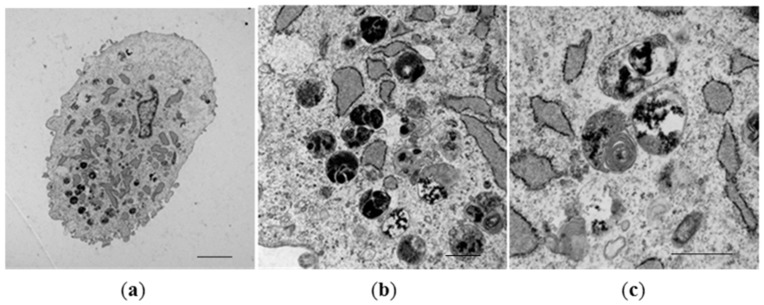
Expression of electron-dense nanoparticles by *mms6*-transfected MSCs. Representative TEM pictures of *mms6*-expressing MSCs cultured for 15 days in medium containing ferric quinate. Scale bars: (**a**) = 10 µm; (**b**) = 100 nm; (**c**) = 500 nm.

**Figure 4:**
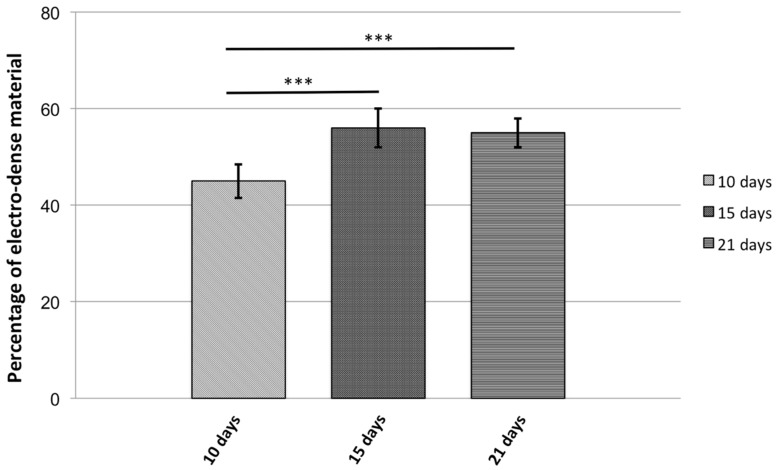
Nanoparticle formation in mms6-transfected MSCs. Percentage of electron-dense material present in intracytoplasmic vesicles at 10, 15, and 21 days in culture. Results are mean ± standard deviation error bars, *n* = 10. *** *p* < 0.05.

**Figure 5:**
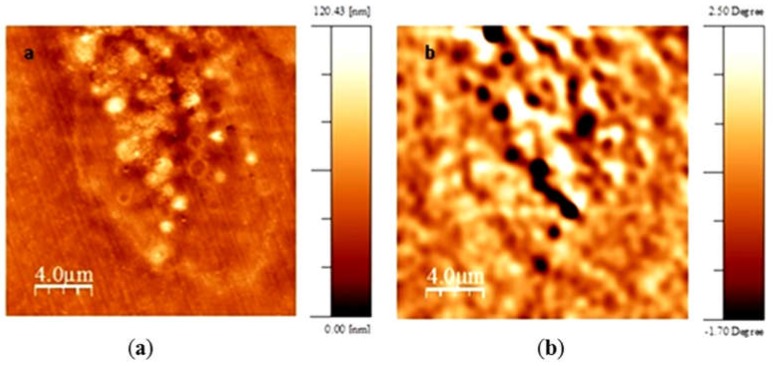
Atomic force microscopy/magnetic force microscopy (AFM/MFM) of 21 days *mms6*-transfected MSCs. Topographic AFM image obtained in semi-contact mode (**a**) and its corresponding MFM phase image (**b**), obtained at 30 nm lift distance. Positive and negative signals contrast corresponding to intracellular aggregates.

RT-PCR confirmed expression of both genes from day 7 to day 21 in culture which was associated with accumulation of membrane-bound, intracytoplasmic electron-dense nanoparticles identified by transmission electron microscopy (TEM) (Figure 3). MSCs expressing *mms6* and *mmsF* genes contain vesicles filled with both dispersed and larger electron-dense material with vesicles measuring up to 1 µm in diameter. Image analysis studies showed an increase in the amount of electron-dense material within cytoplasmic vesicles of *mms6* transfected cells from day 10 to day 15 in culture without an increase in material deposition thereafter (Figure 4). In vitro phantom studies on *mms6*-transfected cells following 21 days in culture in the presence of ferric quinate indicated that cells were identifiable by MRI, confirming previous studies that *mms6* could act as a MR reporter gene. Atomic force microscopy/magnetic force microscopy (AFM/MFM) indicated that the synthesized nanoparticles within *mms6*-transfected cells were magnetic (Figure 5) in contrast to MSCs incubated with FeCl_3_ and untransfected MSCs in which no magnetic structures were identified [48]. To further characterize the magnetic nature of the intracytoplasmic particles, we have used SQUID magnetometry which measures the total magnetic moment of a sample, including all atomic and molecular magnetic contributions. Results of these measurements for MSCs 21 days after transfection confirmed the magnetic nature of the cellular nanoparticles of the nanoparticles synthesized by the *mms6*- and *mmsF*-transfected cells [48]. The *mms6*-transfected MSCs appear to show greater magnetism than *mmsF*-transfected MSCs with *mms6/mmsF* co-transfected MSCs showing least magnetism (unpublished results).

To assess whether *mms6* transfection and induction of magnetic nanoparticle production might have effects on MSC function that would have adversely influenced their ability to aid specialized tissue repair, studies on cell proliferation, migration, and differentiation have been undertaken. No adverse effects on MSC proliferation or migration have been identified. FACS analysis showed no change in phenotype with respect to expression of the cell surface markers ecto-5-prime-nucleotidase/CD73, Thy1-antigen/CD90 or endoglin/CD105 up to 21 days post transfection. (Table 1). When *mms6*-transfected MSCs are cultured for 3 weeks in osteogenic media expressed alpha-1 type I collagen (COL1A1) and bone gamma-carboxyglutamic acid-containing protein (BGLAP) and stained positively with von Kossa stain, it indicates maintenance of osteogenic differentiation potential. Chondrogenic differentiation of transfected MSCs was established by culturing transfected MSCs in chondrogenic media where expression of *COL2A1* and *ACAN* was evident and proteoglycan synthesis was demonstrated, whilst culture in adipogenic media resulted in expression of Peroxisome proliferator-activated receptor gamma (PPARG) and oil-red O staining, indicating that the transfected MSCs maintained the capacity for specific lineage differentiation [48].

**Figure 6:**
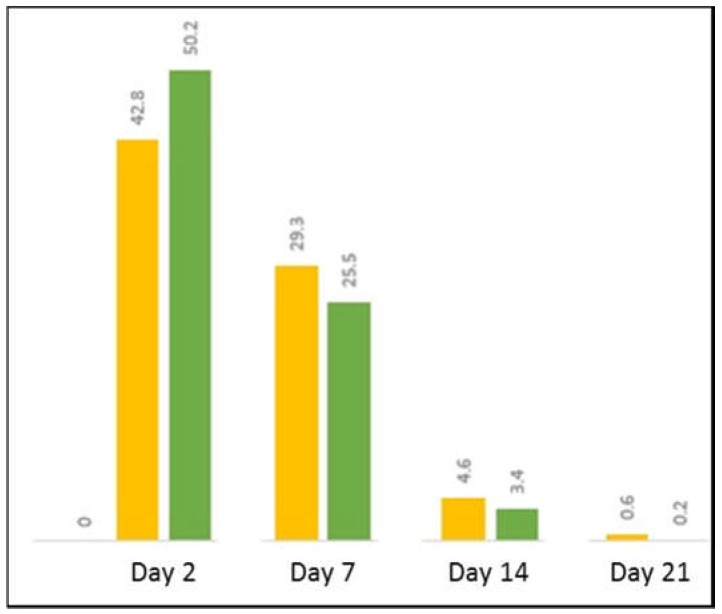
*mms6* expression decreases with time in culture. MSCs were transfected with a pcDNA 3.1 plasmid containing either a wild-type (yellow) or codon-optimized (green) *mms6* gene with an added GFP-tag at the C terminus. The percentage of cells demonstrating GFP fluorescence was assessed by Fluorescence-activated cell sorting (FACS) analysis at different times in culture.

**Figure 7:**
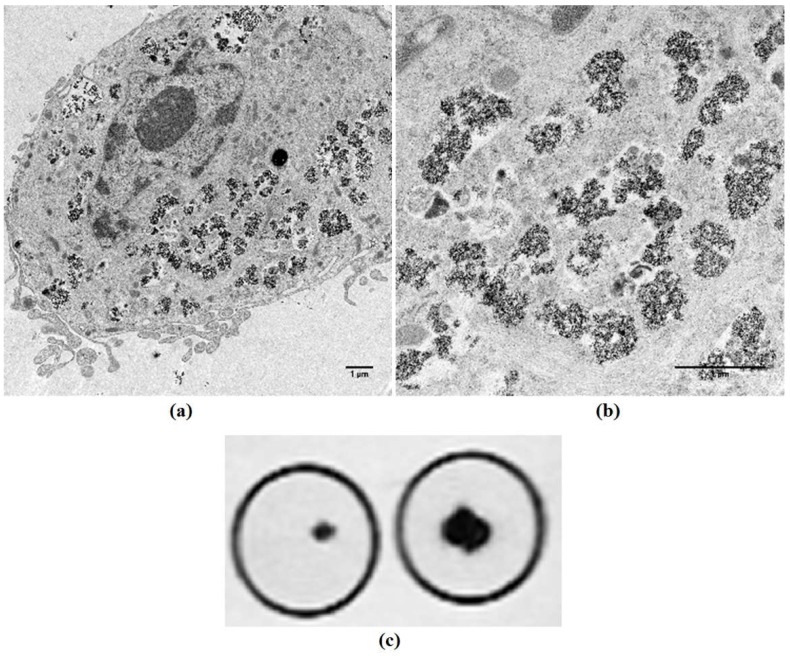
Lentiviral-transduced MSCs express intracellular nanoparticles which are identifiable by MRI. (**a**,**b**) TEM of MSCs transduced with a lentivirus containing a codon-optimized *mms6* gene. Scale bar = 1µm (**c**) magnetic resonance (MR) imaging identifies *mms6*-transduced MSCs (left) and MSCs containing Ferucarbotran magnetic nanoparticles.

These studies have demonstrated proof of concept that MTB magnetosome-associated genes can be expressed in human MSCs but studies with His and Green Fluorescence Protein (GFP)-tagged *mms6* genes show decreased protein production with time in culture, most likely as a consequence of transient gene expression (Figure 6). As such, for longer, in vivo-related studies it is important to develop stable expression cells as has been done for *magA*. This should allow increased gene expression, elevation in protein production, and enhanced formation of magnetic nanoparticles. Continued gene expression in MSC progeny would also maintain cell magnetism and potentially aid the tracking, localization, and retention of magnetic MSCs over a prolonged period. To this end, we have created a lentiviral construct which has allowed stable expression of Mms6 in MSCs. These transduced cells contain intracytoplasmic nanoparticles and can be imaged by MR (Figure 7).

## 4. Tracking, Localization, and Retention of Magnetic MSCs

The self-renewal capabilities and multidirectional differentiation potential of MSCs make them ideal for cell-based therapy in regenerative medicine as they may be induced under different conditions to differentiate into a range of tissues such as bone, cartilage, adipose, muscle, endothelial, and nerve tissue. However, optimal effectiveness of cell-based therapy depends upon MSCs being delivered to and retained at the site of interest where they need to be appropriately induced to differentiate. Although MSCs selectively migrate towards damaged tissue, long-term retention at sites of injury following systemic or local administration of MSCs can be poor [50]. There is therefore an increasing requirement to identify both the fate of the therapeutic cells following delivery and, where possible, retain these cells optimally at the site of tissue damage/repair [51,52,53,54,55]. In vitro labelling of stem cells with magnetic iron oxide particles or by transgenic approaches [55,56] allows in vivo imaging by MR, whereas magnetic targeting (MT) may enhance attraction and retention of labelled cells by application of a magnetic field over the area of interest following either local or systemic injection.

### 4.1. MR Imaging

Magnetic resonance imaging allows high-resolution visualization of appropriately labelled MSCs when delivered in vivo [57]. Several types of contrast agents added to MSCs have been used for MRI in vivo imaging. These include SPIONs but the potential of a transgenic approach to magnetizing MSCs for MR imaging is also under active consideration [56]. Cells transfected with *magA*, cloned from both MS-1 and AMB-1 species of MTB, show increased MR contrast in stably transfected mammalian cells, in response iron supplementation [45,46]. Mms6 has also been studied as a potential reporter gene. Expression in a mammalian cell line allowed differentiation, in vivo, of tumors that stably expressed *mms6* from those that did not, even in the absence of exogenous iron supplementation [47]. Our in vitro results with transgenic MSCs confirm that *mms6* can function as a MR reporter gene with the potential to allow MR tracking in vivo [48]. 

### 4.2. Magnetic Targeting

The type of magnetic device used to target MSCs will change depending on the body location to which the cells are to be targeted. Magnetic targeting relies on magnetization of MSCs followed by in vivo targeting with the aid of magnetic fields with the aim to allow and retain the maximum number of cells administered to reach the site of required repair, allowing limitation of the cell number and volume injected [58]. Typically, this may be with an external magnet [59,60] but increasingly magnetic implants are being developed, as in the orthopedic field where insertion of magnetic scaffolds with or without permanent magnets can be used to aid healing of massive bone defects and chondro-osseous defects [61]. In vivo guidance of the magnetized MSCs by external static magnetic fields for magnetic targeting can be through the use of permanent magnets or electromagnets, although permanent magnets are likely to be preferred since they are portable, reach higher field strengths compared to electromagnets of similar size, and do not require a power supply or cooling system. Permanent magnets are normally placed externally, with multiple magnets sited in different positions in relation to the area of interest, allowing a static magnetic field to be extended to deeper parts of the body. The use of external magnets for MT is now reaching the clinic [62,63]. Kamei et al. [63] used autologous bone marrow MSCs magnetized with Ferucarbotran with an external 1.0 Tesla compact magnet for treatment of knee joint cartilage defects in 5 patients. 1 × 10^7^ MSCs were injected into the knee joint and the magnet was maintained in the same position for 10 min after the MSC injection. There were no significant initial adverse effects and outcomes, with respect to cartilage repair and clinical outcome, after 48 weeks were good. While external magnets avoid the risk of an inflammatory response to the inserted materials, proof-of-principle studies have indicated that implanted permanent magnets and scaffolds have potential in a range of settings including bone repair [61,64,65,66]. In addition to enhancing targeting and retention of magnetic MSCs, magnetic fields may directly enhance MSC functionality. In vitro studies have demonstrated enhanced cell adhesion, viability, proliferation, secretory activity, as well as differentiation potential [67,68,69] when MSCs are exposed to an external magnetic field, effects which appear in part to be mediated through a phosphoinositide 3-kinase/Akt signaling pathway [70].

## 5. Magnetic MSCs and Hyperthermia

Hyperthermia is a therapy in which heat is increased within a tumor to an extent that elevation of the local temperature into a range of 41–56 °C induces a range of cellular changes including protein denaturation, damage to the cytoskeleton, disruption of DNA repair which lead to cell death via apoptosis or necrosis [71,72,73]. Other beneficial effects include stimulation of the immune system [74]. The use of hyperthermia for cancer treatment has benefits in that the toxic side effects are less than that of chemotherapy and radiotherapy. Furthermore, cancer cells are more sensitive to increases in temperature than the surrounding healthy cells, a characteristic which may relate to altered vasculature within tumors [75,76]. 

A number of different techniques have been used to induce hyperthermia, including application of microwaves, radio waves, or ultrasound [77], but there is an increasing interest in the development and use of magnetic hyperthermia (MH) therapy for a number of difficult-to-treat cancer types such as glioblastoma and in the management of a diverse range of primary, recurrent, and metastatic tumors that are not amenable to standard surgical, radio- or chemotherapy treatment [78,79]. MH is based on the concept that MNPs generate heat when exposed to an external alternating magnetic field (AMF). There are two mechanisms recognized as being responsible for heat production by MNPs in an external magnetic field. These are the magnetic hysteresis loss, occurring in particles with multimagnetic domains, and Néel and Brownian relaxation that are present in superparamagnetic or single-domain particles [80]. Local hyperthermia is thereby produced due to the rotation of the nanoparticles (Brownian relaxation) and flipping of the magnetic dipoles due to rotation of the magnetic moments in the magnetic field (Néel relaxation) [81]. Intratumoural-injected MNPs generate heating effect after application of an external magnetic field and induce cell death [82,83]. MNPs used for MH are typically metals such as iron, cobalt, and nickel and metal oxides including Fe_3_O_4_, γ-Fe_2_O_3_, MnFe_2_O_4_, and CoFe_2_O_4_. However, as degradation of MNPs within the body results in the production of reactive oxygen species (ROS) and free radicals which trigger lipid peroxidation, DNA damages, protein alteration, and cell apoptosis, MNPs are usually subjected to a range of surface modifications that increase stability and safety. These include coating with dimercaptosuccinic acid (DMSA), polyethylene glycol (PEG), dextran, chitosan, liposomes, gold, or silica, which function to decrease agglomeration, increase biocompatibility and pharmacokinetics [84].

Magnetosomes have also been studied for MH treatment of tumors [2]. Application of an AMF of frequency 183 kHz and field strengths of 20, 40, or 60 mT to the MDA MB 231 breast cancer cell line to which extracted chains of magnetosomes had been added induced up to 100% cell death of these cells in vitro. Importantly, similar efficacy was seen in vivo when three 20 min cycles of MH were applied following injection of a suspension containing ∼1 mg of extracted chains of magnetosomes into a tumor arising following subcutaneous inoculation of the breast cancer cell line [85]. In this study, tumor regression was seen with elevation of local temperature to 43 °C. Recently, magnetosomes which have been made biocompatible by removing potentially toxic organic bacterial residues and by coating with poly-l-lysine, leading to magnetosomes-poly-l-lysine have been used in MH preclinical studies [86]. Injection of 25 µg iron of nanoparticles per mm^3^ of tumor and application of 11 to 15 30 min sessions of 198 kHz, 11 to 31 mT, AMF resulted in significant longer-term survival in around 50% of mice. 

Challenges limiting the clinical use of MH, however, include obtaining adequate and reproducible localization of MNPs to the tumor site and improving the specific heating power of MNPs in order to use the minimum required amount of MNPs [87]. MNP administration has traditionally been by direct intratumoural injection or through an intravenous route. Direct intratumoural injections have the advantage of achieving high local concentrations of MNPs and limiting systemic toxicity. Disadvantages of this approach include inhomogeneous intratumour distribution potentially preventing complete eradication of tumors following induction hyperthermia and its non-applicability to treatment of multifocal metastatic disease. Although intravenous administration has the potential to cover irregular tumor shapes more precisely, MNPs are rapidly removed from the blood stream by the reticuloendothelial system and doses required to lead to significant loading of tumor tissue for induction hyperthermia tend to be systemically toxic. Locally injected MNPs also redistribute following administration. To overcome at least some of these problems, the idea of using cells as carriers of MNPs is gaining acceptance. When RAW264.7 mouse monocyte/macrophage-like cells were loaded with iron oxide nanoparticles and injected into the peritoneum of mice bearing pancreatic tumors, there was preferential homing of the MNP-bearing cells to the tumors with limited spread to normal organs [88]. There is now compelling evidence that MSCs can be utilized as delivery vehicles for cancer therapeutics taking advantage of their innate tropism to the tumor site and their low immunogenicity. Human MSCs administered through a tail vein have been shown to preferentially engraft at tumor sites [89]. Observations that some studies have failed to detect MSC tumor-homing from intravenous injection whereas extensive MSC migration within gliomas following intratumoural injection indicates that direct in situ administration of MNP-bearing MSCs may have potential in the clinical setting for MH [90]. It remains unclear whether MSCs can be used as a delivery system for MNPs in cancer treatment. A recent study failed to show a significant differences in overall tumor size or growth characteristics in a mouse model using Ferucarbotran-loaded human MSCs despite labeled MSCs being incorporated into and retained within the tumors [91]. Although magnetosomes have been shown to have a magnetic hyperthermia effect, the efficiency magnetosome chains appear to be greater in the killing of tumor cells than individual, separated magnetosomes due to better distribution within cells [90]. It remains to be seen whether genetically modified MSCs are able to produce magnetic nanoparticles in sufficient amount and in an appropriate configuration to act as suitable agents for MH.

## 6. Conclusions

The use and potential of magnetic MSCs in tissue engineering is continually evolving with necessary advances in cell magnetization technology. MSCs are able to be magnetized by the addition of MNPs to cell cultures or through a transgenic approach using magnetosome-related genes from magnetobacteria. A number of studies have now shown that magnetotactic bacterial genes are expressed in mammalian systems and expression is associated with magnetic nanoparticle production allowing MR imaging. Whether the transgenic expression of magnetic nanoparticles allows magnetic targeting and what the long-term effects of these approaches on MSC function in vivo may be, however, need to be understood. Certainly, improved methods of magnetic targeting of magnetized stem cells through the use of external and internal magnets or magnetic scaffolds are likely to be essential for optimal targeting and retention of magnetic MSCs for enhanced tissue repair.

## Figures and Tables

**Table 1:** Expression of MSC differentiation antigens following 21 days in culture. Wild-type or transfected MSCs were cultured in the absence or presence of ferric quinate.

Differentiation Antigen	Non-Transfected MSCs	Non-Transfected MSCs with FeQ	*mms6*-Transfected MSCs with FeQ
CD34	3.1 ± 0.1	2.9 ± 0.3	3.2 ± 0.2
CD73	99.9 ± 0.1	99.8 ± 0.1	99.8 ± 0.1
CD90	99.9 ± 0.1	99.6 ± 0.1	98.1 ± 0.1
CD105	98.8 ± 0.1	98.7 ± 0.1	98.3 ± 0.1
